# Nutritional Quality Fingerprinting of Wild and Farmed *Cyprinus carpio*: A UHPLC-MS/MS-Based Traceability Strategy

**DOI:** 10.3390/biology14121695

**Published:** 2025-11-28

**Authors:** Lang Zhang, Wenya Ji, Wenwen Suo, Ziwei Song, Wei Yang, Xinbin Duan, Jizhou Lv, Lei Gao, Liting Ye, Zhen Li, Yali Yu, Hui Zhang

**Affiliations:** 1 Yangtze River Fisheries Research Institute, Chinese Academy of Fishery Sciences, Wuhan 430223, China; zhanglang@yfi.ac.cn (L.Z.); 2024308120031@webmail.hzau.edu.cn (W.J.);; 2 College of Fisheries, Huazhong Agricultural University, Wuhan 430070, China; 3 Hunan Fisheries Research Institute and Aquatic Product Seed Stock Station, Changsha 410153, China; 4 Key Laboratory of Hydraulic and Waterway Engineering of the Ministry of Education, College of River and Ocean Engineering, Chongqing Jiaotong University, Chongqing 400074, China; 5 Chinese Academy of Inspection and Quarantine, Beijing 100176, China

**Keywords:** Yangtze River, UHPLC-MS/MS, metabolomics, ecological restoration, *Cyprinus carpio*

## Abstract

In the context of the ten-year fishing ban on the Yangtze River, illegal poaching for profit persists. To support the enforcement of this ban and protect the river’s ecosystem, an efficient and precise method for distinguishing between wild and farmed common carp is essential. This study utilized ultra-high-performance liquid chromatography-tandem mass spectrometry (UHPLC-MS/MS) combined with metabolomics technology to analyze and compare the metabolic differences between wild and farmed common carp. Principal component analysis (PCA) and orthogonal partial least squares discriminant analysis (OPLS-DA) revealed a clear separation between the two groups, which was further verified by metabolic fingerprint profiles. Moreover, 16 metabolites with high discriminatory potential were identified from 491 differentially metabolites, such as phytosphingosine, succinic acid and threonine. In addition, a cluster analysis of the differential metabolites classified them into four classes: peptides, fatty acyls, steroids and steroid derivatives, and glycerophospholipids. Furthermore, candidate biomarkers, including 3-hydroxybutyrylcarnitine, 3-hydroxyhexanoylcarnitine and jasminoside were identified to potential distinguish wild populations. To our knowledge, this is the first study to apply metabolomics technology to differentiate wild from farmed common carp, providing a new theoretical basis for ecological restoration efforts in the context of the Yangtze River fishing ban.

## Simple Summary

To restore ecological balance and sustain fishery resources, a ten-year fishing ban was implemented in the Yangtze River to reduce human impact and support fishery recovery. However, illegal poaching persists, and traditional morphological methods struggle to distinguish between wild and farmed common carp (*Cyprinus carpio*). Our study compared and analyzed the metabolites in the muscle of wild and farmed common carp in the Yangtze River Basin. Wild common carp have increased purine metabolism substances like xanthine and uric acid, while farmed common carp have higher zearalenone levels, likely due to environmental instability and feed factor. These findings offer potential biomarkers to differentiate between wild and farmed common carp, aiding in efforts to combat illegal fishing.

## 1. Introduction

The Yangtze River, China’s important freshwater fishery resource, plays a critical role in maintaining ecological balance and supporting biodiversity. However, these freshwater ecosystems are vulnerable to various human activities. For instance, reservoirs affect aquatic biodiversity by disrupting organism movement, flow regimes, and habitats [1]. In addition, intensive human activities such as dam construction, land reclamation, overfishing have severely degraded the Yangtze River’s ecology, disrupting habitats, and causing sharp declines in fish biodiversity and fishery resources [2]. Survey data illustrates that while the fish catch in the mid-lower Yangtze River and its tributaries peaked at 427,000 tons in 1954, it plummeted by nearly 85% to a mere 66,000 tons by 2016 [3]. To restore ecological balance and ensure the sustainability of fishery resources, a ten-year fishing ban was implemented in the Yangtze River, commencing on 1 January 2020 [4]. This ban aims to mitigate the impacts of human activities and promote the recovery of fisheries.

After the fishing ban was implemented, illegal poaching has persisted [5]. Various factors contribute to the spread of illegal fishing, but the actions are consistently linked to high profitability [6]. The common carp is a common edible fish that may enter markets through illegal fishing activities, making it challenging for consumers to identify its origin [7]. This phenomenon has caused significant disruption to market governance, diminishing the effectiveness of the ten-year fishing ban’s implementation [8]. Consequently, establishing an efficient, convenient, and accurate method for discriminating between farmed and wild common carp is crucial for enhancing consumer market regulation.

Accurately determining whether fish are wild-caught or farmed, along with their geographical origins, is crucial for ecological management, consumer trust, and the enforcement of regulations [9,10]. Traditional morphological methods are capable of differentiating between wild and farmed common carp; however, these techniques frequently lack accuracy and pose significant challenges in their implementation. Previous studies have indicated that farmed common carp exhibit fewer lateral line scales, caudal fin rays, and vertebrae, but more dorsal and anal fin rays [11]. Morphological methods not only require high professional expertise from the identifiers but are also susceptible to subjective influences, resulting in relatively low accuracy [12,13]. In addition, a study conducted genetic diversity analysis of wild common carp from three distinct Turkish lakes using microsatellite markers, revealing a much higher genetic variability of the three Turkish wild common carp populations compared to domesticated/captive stocks [14]. This method is highly accurate but is primarily suited for macro-scale genetic structure analysis, demonstrating limited efficacy in the precise discrimination between wild and cultured individuals. Furthermore, stable isotope analysis can identify the origin of certain wild-caught fish species, but its use is complicated by the need for timely sampling and seasonal changes in food web structure and nutrient flow, which can cause isotopic shifts [15,16]. Although each method has been proven valuable, they face specific limitations. In addition to these tools, metabolomics has garnered significant attention due to its high sensitivity and specificity, enabling the detection of subtle differences that traditional physiological parameters may not identify [17]. In the context of distinguishing between wild and farmed fish, the application of metabolomics has been comparatively infrequent, yet it presents innovative potential for advancing traceability research. Nevertheless, metabolomics is most effective when combined with other methods, enhancing accuracy and reliability in various fields and advancing scientific research and applications [18,19,20].

Differences in living environments and dietary habits between wild and farmed fish may lead to distinct metabolic profiles, a hypothesis supported by previous studies [21,22]. The accurate discrimination between wild and farmed common carp needs highly sensitive analytical methods. UHPLC-MS/MS has been widely applied in the fields of water pollutant detection and agricultural product residue testing. For instance, a study demonstrated that a semi-automated high-throughput approach combining fast LP-GC-MS/MS and UHPLC-MS/MS could effectively analyze 302 pesticide and environmental contaminant residues in catfish [23]. Similarly, UHPLC-MS/MS has been employed to detect fluorinated emerging pollutants in shrimp aquaculture systems [24], demonstrating its effectiveness in identifying and quantifying contaminants that can impact aquatic food safety. Despite its common use in aquatic studies for its high sensitivity and accuracy [25,26], its potential in metabolomics analysis for tracing the biological origins of aquatic animals, such as differentiating wild from farmed fish, is still underutilized.

This study represents the first application of UHPLC-MS/MS-based metabolomics to identify potential metabolic biomarkers discriminating wild from farmed common carp. This study aims to develop a high-efficiency and precise discrimination technique for wild and farmed common carp. The findings of this research contribute to equitable market regulation and support the implementation of a ten-year fishing ban on the Yangtze River.

## 2. Materials and Methods

### 2.1. Collection and Preparation of Test Samples

**Figure 1:**
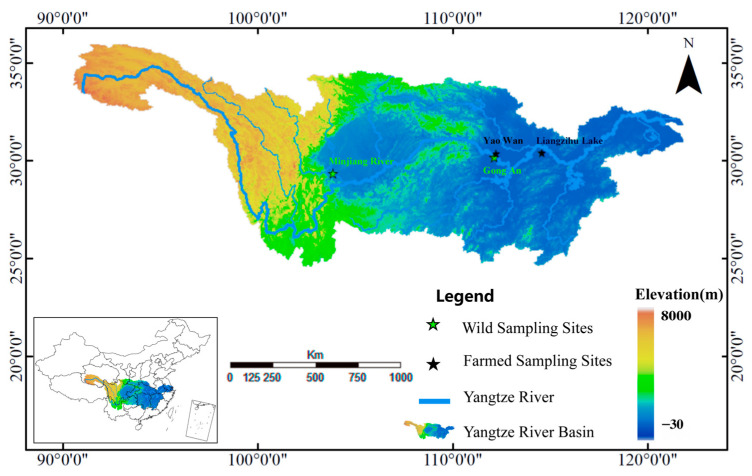
Geographical location map of sampling sites for wild common carp and farmed common carp.

Following the implementation of the Yangtze River fishing ban, all productive fishing activities have been prohibited. In this study, sample collection from Yangtze River was conducted under a scientific fishing permit. To eliminate potential confounding effects due to sex differences, only male common carp were selected as experimental subjects. Males were reliably identified by their prominent nuptial tubercles (breeding stars) [27]. To ensure accuracy, a random subset was further verified as male through gonadal examination post-sacrifice. To ensure sample diversity, this study selected 18 male common carp from each of the four sites shown in Figure 1 (coordinates based on WGS1984, EPSG:4326; map generated using ArcGIS version 10.8 by Esri, Redlands, CA, USA), totaling 72 specimens. From 1 May to 30 June 2023, farmed common carp were collected from Liangzi Lake Aquaculture Base (farmed-1) and Yao Wan Aquaculture Base (farmed-2), while wild common carp were captured in the Yangtze River at Minjiang River (wild-1) and Gong An (wild-2). Fish sampling was performed utilizing a three-layer fixed gillnet with an inner mesh size of 70 mm, in strict accordance with established scientific protocols. To minimize the potential confounding effects of body weight on metabolic differences, individuals with similar body weights were selected for subsequent analysis. The mean weights of each group were as follows: farmed-1 (2385.46 ± 118.74 g); farmed-2 (2437.15 ± 107.63 g); wild-1 (2512.93 ± 132.85 g); and wild-2 (2489.72 ± 141.56 g).

The euthanasia and sampling protocol was adapted from a previous study [28]. Fish were deeply anesthetized by immersion in 250 mg/L MS-222 for 30 s, and then the gill arch was transected. Dorsal skeletal muscle tissue was then immediately collected from both sides, flash-frozen in liquid nitrogen, and stored at −80 °C for subsequent metabolomic analysis.

The sampling methodology for the water samples is informed by prior research [29]. Sampling was conducted using pre-treated 500 mL polyethylene bottles. Before sampling, the bottles were rinsed three times with deionized water and three times with the test water. The sealed bottle was inverted and submerged 10–15 cm below the water surface, facing the flow direction. After opening the cap, the bottle filled naturally. Three parallel samples were collected at each site and immediately placed in a 4 °C preservation box for transport.

### 2.2. Untargeted Metabolomics via UHPLC-MS/MS

Untargeted metabolomic analysis was performed following established method [30]. For subsequent UHPLC-MS/MS analysis, 18 individual muscle samples from each region were randomly divided into 6 groups, and 3 samples were conducted for each group. The composite samples were flash-frozen in liquid nitrogen, homogenized by cryogenic grinding, aliquoted, and stored at −80 °C, yielding 6 biological replicates per region (n = 6). In prior untargeted metabolomics research, the number of replicates generally ranged from three to six to ensure data stability and reproducibility [17,30]. Samples were thawed at 4 °C, and 50 mg of each sample was weighed. Two steel beads were added to each sample along with 400 μL of pre-chilled methanol-water solution (4:1, *v*/*v*; methanol). The samples were homogenized at low temperature using a Vortex Mixer (QL-866, Kylin-Bell Lab Instruments Co., Ltd., Haimen, China). Then, 600 μL of pre-chilled methanol-water solution (4:1, *v*/*v*) was added and mixed thoroughly. The mixtures were sonicated in an ice bath for 20 min and kept at −20 °C for 1 h. After centrifugation at 16,000× *g* for 20 min at 4 °C, the supernatants were collected and dried using a high-speed vacuum centrifugal concentrator (Eppendorf, Concentrator plus, Hamburg, Germany). Mass spectrometry was performed with a Q Exactive Plus mass spectrometer (Thermo Scientific, Waltham, MA, USA), the extracts were reconstituted in 100 μL of pre-chilled methanol-water solution (1:1, *v*/*v*), centrifuged at 20,000× *g* for 15 min at 4 °C, and the supernatant was collected for injection analysis. A pooled quality control (QC) sample was analyzed throughout the sequence to monitor stability.

For chromatographic separation, the samples were maintained at 4 °C in the autosampler throughout the entire analytical process. The separation was performed using a SHIMADZU-LC30 UHPLC system equipped with an ACQUITY UPLC^®^ HSS T3 column (2.1 × 100 mm, 1.8 µm; Waters, Milford, MA, USA). The injection volume was 4 µL with the column temperature set at 40 °C and a mobile phase flow rate of 0.3 mL/min. The mobile phase consisted of 0.1% formic acid (St. Louis, MO, USA) in water (A) and 0.1% formic acid in acetonitrile (B). The gradient elution program was as follows: 0–2 min, 0% B; 2–6 min, linear increase from 0% to 48% B; 6–10 min, linear increase from 48% to 100% B; 10–12 min, 100% B maintained; 12–12.1 min, linear decrease from 100% to 0% B; and 12.1–15 min, B was maintained at 0%.

During mass spectrometry acquisition, each sample was analyzed using electrospray ionization (ESI) in both positive (+) and negative (−) ion modes. Following UHPLC separation, the samples were subjected to mass spectrometric analysis using a QE Plus mass spectrometer (Thermo Scientific, Waltham, MA, USA) equipped with a HESI ion source. The ionization parameters were set as follows: Spray Voltage: 3.8 kV (+) and 3.2 kV (−); Capillary Temperature: 320 °C; Sheath Gas: 30 arbitrary units; Auxiliary Gas: 5 arbitrary units; Probe Heater Temperature: 350 °C; S-Lens RF Level: 50. The mass spectrometry acquisition was performed with the following parameters: a total acquisition time was set at 15 min, with a precursor ion scan range of 75–1050 *m*/*z*. The MS1 resolution was 70,000 at *m*/*z* 200, with an AGC target of 3 × 10^6^ and a maximum injection time of 100 ms. For MS2 analysis, the top 10 most intense precursor ions from each full scan were selected for fragmentation. The MS2 scanning was conducted at an *m*/*z* value of 200, employing a resolution of 17,500. The AGC target was configured to 1 × 10^5^, with a maximum injection time of 50 milliseconds. Fragmentation was executed utilizing higher-energy collisional dissociation (HCD) technology, with parameters set to an isolation window of 2 *m*/*z* and stepped collision energies of 20, 30, and 40.

### 2.3. Metabolite Data Preprocessing and Screening

The data analysis methods referred to previous research [30]. Raw data were processed using MSDIAL ver.4.9.221218 for peak alignment, retention-time correction, and peak-area extraction. Metabolite identification was performed by accurate-mass matching (mass tolerance < 10 ppm) and MS/MS spectral matching (mass tolerance < 0.01 Da) against public databases (Human Metabolome Database (HMDB), MassBank, and the Global Natural Products Social Molecular Networking (GNPS) platform) and an in-house BP-DB metabolite standard library. Subsequently, features with more than 50% missing values within any group were removed. Positive-ion and negative-ion data were normalized by total peak area, merged, and imported into Python 3.0 for pattern recognition. After Unit Variance (UV) scaling, the dataset was subjected to further statistical analyses.

The pre-processed peaks list was subjected to multivariate statistical analysis using SIMCA (version 14.1) (Umetrics, Umeå, Sweden). Initially, unsupervised PCA was conducted to preliminarily observe sample distribution patterns, followed by the construction of a supervised OPLS-DA model. Prior to analysis, the data underwent standardization and Pareto scaling treatment. Model quality was evaluated through the model explanation rate (R^2^Y) and cross-validated predictive ability (Q^2^). To verify the reliability of the OPLS-DA model, permutation testing with 200 iterations was performed to exclude overfitting risks.

### 2.4. Screening and Identification of Candidate Biomarkers

The screening of differential metabolites was based on Variable Importance in Projection (VIP) values, *p*-value, Fold Change (FC), and Area Under the Curve (AUC) values. VIP values were calculated using OPLS-DA, followed by a Wilcoxon test with FDR correction for significance. FC values were computed as an effect size. Variables with VIP > 1, *p*-value < 0.05 and |log_2_(FC)| no less than 1 were determined as differentially expressed [31]. Dynamic receiver operating characteristic (ROC) analysis on the Genedenovo (https://www.genedenovo.com/; accessed on 25 June 2025) platform was used to select substances with suitable AUC values for metabolite category differentiation. The selection of the most promising candidate biomarkers was performed using ROC curve analysis. The selection criteria included an AUC > 0.9, ensuring the reliability and significance of the results [32].

### 2.5. Statistical Analysis

Data were analyzed using SPSS 22.0 (SPSS Inc., Chicago, IL, USA). Inter-group comparisons were performed by one-way analysis of variance (ANOVA), where results were expressed as mean ± standard deviation and statistical significance was set at *p* < 0.05, while multivariate statistical analysis of the pre-processed peaks list was conducted in SIMCA (version 14.1) (Umetrics, Umeå, Sweden) software through PCA and OPLS-DA. Data visualization, including UHPLC-MS/MS fingerprints of characteristic metabolites, boxplots, and interval graphs, was generated using Origin (version 2024) (OriginLab, Northampton, MA, USA) Software, along with volcano plots and heatmaps created using the Genedenovo (https://www.genedenovo.com/; accessed on 28 June 2025) and Personalbio (https://www.genescloud.cn/login; accessed on 28 June 2025) platforms.

### 2.6. Determination and Analysis of Water Samples

The determination of water quality indicators is conducted using the following methodologies: Total Phosphorus (TP) is assessed via Ammonium Molybdate Digestion-Ultraviolet Spectrophotometry [33]; Metal Elements, including Copper (Cu), Calcium (Ca), and Magnesium (Mg), are quantified through direct determination using Inductively Coupled Plasma Optical Emission Spectrometry (ICP-OES) [34]; and Ammonia Nitrogen (NH_4_-N) is measured by Alkaline Potassium Permanganate Digestion-Ultraviolet Spectrophotometry following filtration through a 0.25 μm filter membrane [35]. Regarding sample processing, six sample groups are combined into three composite samples through random pairwise mixing, while three sample groups remain unchanged. All samples, along with triplicate parallel water quality data, are subsequently analyzed using the GenesCloud platform for Redundancy Analysis (RDA).

## 3. Results and Discussion

### 3.1. Differentiation of Common Carp Samples from Wild Sections and Farmed Sections

#### 3.1.1. PCA and OPLS-DA

QC samples into the metabolomic analysis of this study enabled the assessment of data consistency and stability across both positive and negative ion modes. As shown in Figure S1, the findings revealed that the correlation coefficients (Corr) among the QC samples exceeded 0.99, thereby indicating a high degree of reproducibility and reliability in the experimental data. To conduct a preliminary investigation into the differences in the metabolome between the farmed and wild groups, as well as to assess the parallelism of samples within each group, hierarchical clustering analysis was applied. As shown in Figure S2, good parallelism among samples within each group, demonstrating the reproducibility of the findings. Moreover, the clustering dendrogram demonstrated a distinct grouping trend between samples from the different groups, indicating preliminary differences in metabolomic characteristics between the farmed and wild groups.

**Figure 2:**
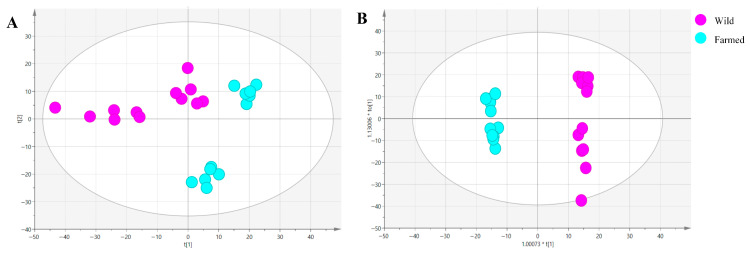
(**A**) shows the PCA score plot of compounds from wild and farmed common carp, while (**B**) displays the permutation test results of the OPLS-DA model.

As shown in Figure 2A, unsupervised PCA revealed a clear separation between wild and farmed common carp, indicated significant metabolic differences between these groups. To further corroborate the observed metabolic disparities, we performed supervised OPLS-DA (Figure 2B), which consistently revealed a clear separation, corroborating the distinct metabolic profiles distinguishing wild from farmed common carp. The high values for model parameters, R^2^Y (0.947) and Q^2^ (0.874) exceeded the accepted threshold (R^2^Y > Q^2^ > 0.50) for model fit and predictive ability [36]. The model validation results show that the model is both effective and reliable, and can roughly distinguish between wild and farmed common carp.

#### 3.1.2. Metabolic Fingerprint Profiles Analysis

**Figure 3:**
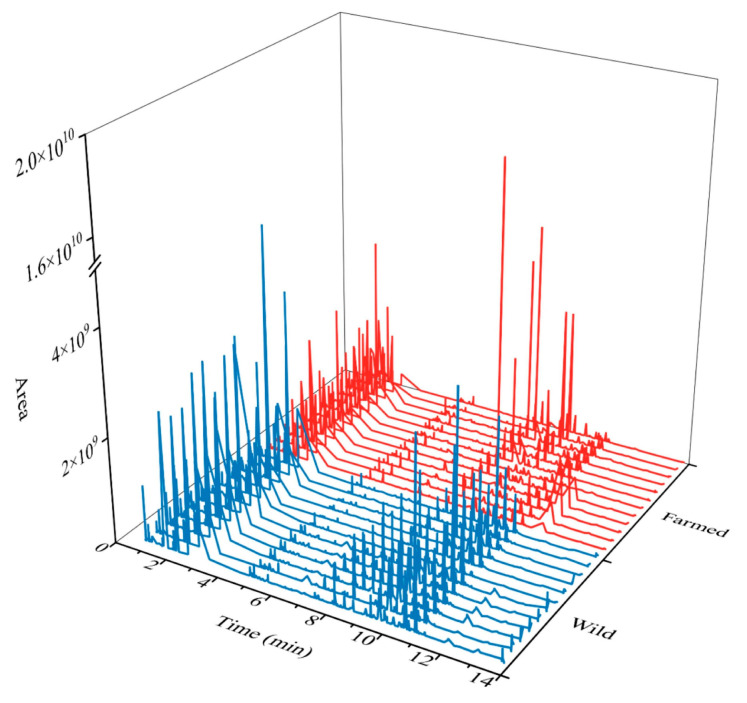
Metabolic fingerprint profiles analysis of the wild versus the farmed common carp. The vertical and horizontal axes display metabolite abundance (Area) and time (min), respectively, with wild and farmed samples colored blue and red.

Fingerprint profiling has demonstrated efficacy in food authentication, such as the identification of yak milk, by enabling the macroscopic recognition of dominant ions [37]. This established methodological reference provides a robust reference for analyzing metabolic differences between wild and farmed common carp. Based on the OPLS-DA results, 491 differential metabolites were identified between wild common carp and farmed common carp using the thresholds: VIP > 1, |log_2_(FC)| ≥ 1, and *p* < 0.05. The characteristic fingerprint profiles were subsequently constructed from these metabolites. As shown in Figure 3, significant differences in metabolic peaks were observed between the two groups. These distinct analytical features demonstrated significant metabolic composition disparities, likely attributable to their differing feeding habits. Existing study indicated that metabolomics fingerprinting can distinguished fish by their feeding habits [38].

### 3.2. Identifying Potentially Discriminated Metabolite Biomarkers in Muscle

**Figure 4:**
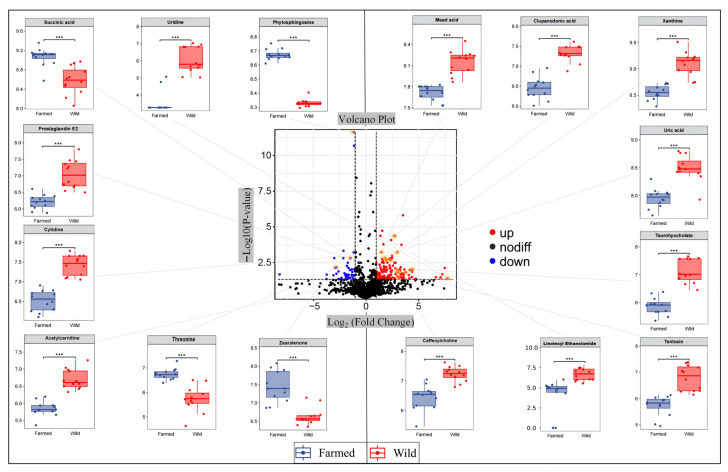
The volcano plot displays differential metabolite profiles between wild and farmed common carp, with candidate biomarkers highlighted in red/blue (black dots denote non-significant metabolites). ROC analysis identified 16 significant putative identifications (AUC > 0.9, indicated by arrows), whose expression levels were visualized via boxplots generated from raw data. *** *p* < 0.001.

Untargeted UHPLC-MS/MS analysis initially detected 1642 metabolites. To identify differential metabolites between wild and farmed common carp, the following criteria were selected: VIP > 1, |log_2_(FC)| ≥ 1, and *p* < 0.05. This process identified a total of 491 metabolites, including 189 down-regulated and 302 up-regulated metabolites. To further explore the key differential metabolites, we performed ROC analysis and identified 16 high-confidence putative identifications (AUC > 0.9) that could distinguish wild from farmed common carp (Figure 4).

The farmed group demonstrated significantly higher levels of 4 putative identifications compared to the wild group: phytosphingosine, succinic acid, threonine, and zearalenone (*p* < 0.05). Numerous studies have indicated that in modern intensive aquaculture, high stocking density is often accompanied by a decrease in water dissolved oxygen (DO), and even hypoxia [39,40], which may be the primary factors contributing to the accumulation of phytosphingosine and succinic acid in farmed common carp. Existing studies have demonstrated that high stocking density induce stress responses in aquaculture species, leading to oxidative damage that compromises physiological functions [41], and may ultimately trigger apoptosis [42]. Phytosphingosine is an apoptosis inducer and can inhibit cell proliferation [43]. This may imply that high-density aquaculture environments lead to metabolic and apoptotic pathway changes in farmed common carp, and affect the content of phytosphingosine. Succinic acid is an important intermediate product in the tricarboxylic acid (TCA) cycle within mitochondria that plays a multifaceted role in the regulation of energy homeostasis [44]. Its accumulation in farmed common carp may be attributable to hypoxic aquaculture conditions, which can impair TCA cycle flux and lead to the pooling of this intermediate metabolite [45]. Moreover, the elevated levels of the essential amino acid threonine may be explained by the high dietary concentration in artificial feed [46]. Zearalenone is a type of mycotoxin usually produced when corn and wheat by-products are contaminated by *Fusarium* fungi [47]. Consequently, it is hypothesized that the elevated levels of zearalenone observed in farmed common carp may originate from the feed used.

In contrast, there was a significantly higher content of 12 putative identifications in the wild group, including xanthine, uric acid, uridine, and other compounds. Specifically, in the purine metabolism pathway, xanthine serves as a key intermediate and is ultimately converted into uric acid [48], thus generating excessive reactive oxygen species (ROS) and hydrogen peroxide [49]. Elevated levels of xanthine and uric acid may indicate an enhancement of the purine metabolic pathway. Previous studies have demonstrated that the purine metabolism mechanism may play a crucial role in balancing ATP demand and supply, thereby enabling shrimp to tolerate environmental or pathogenic stress [50]. Lowering uric acid and hypoxanthine levels may indicate redox balance regulation in fish cells in cold environments, potentially reducing oxidative stress and protecting cells from damage [51]. The observed variations in xanthine and uric acid levels could potentially reflect an adaptive response of common carp to natural aquatic environments, although further studies are needed to confirm this relationship. In addition, clupanodonic acid as a reservoir is metabolized into docosahexaenoic acid (DHA), and further retro-conversed back to eicosapentaenoic acid (EPA) [52]. The existing literature indicates that farmed fish typically contain elevated levels of EPA and DHA relative to wild-farmed specimens, a phenomenon largely governed by the customizable fatty acid profile of aquafeeds [53]. These findings suggest that natural aquatic environments may not provide carps with consistent access to lipid-rich diets comparable to aquafeed formulations, consequently limiting their EPA and DHA metabolic efficiency.

Prostaglandin E2 (PGE2) and tentoxin are both found in higher concentrations in wild common carp. Previous studies have demonstrated that PGE2 modulates immune responses in oysters by activating the E-type prostaglandin receptor (EP4), thereby suppressing the expression of pro-inflammatory cytokines [54]. Based on this, higher PGE2 levels in wild common carp may indicate enhanced immune system modulation. These findings indicate that the comparatively unstable natural environment of wild populations subjects them to greater environmental stress, potentially influence the immune response and the content of PGE2. The detected tentoxin, a cyclic peptide metabolite produced by plant pathogenic fungi [55]. Although the precise mechanism for tentoxin accumulation in wild common carp is currently unknown, it is speculated to originate from plant matter, enter the hydrosphere through runoff or decay, and accumulate in fish tissues.

Cytidine is the precursor of uridine [56], which is involved in multiple biological processes such as carbohydrate metabolism and lipid metabolism [57]. A higher content of cytidine and uridine may indicate that wild common carp have stronger carbohydrate and lipid metabolic capabilities. Previous research has demonstrated that wild populations possess the capacity for rapid evolutionary adaptation in carbohydrate metabolism when subjected to radical environmental alterations [58]. However, research on cytidine and uridine in common carp lipid metabolism is not yet in-depth. We can infer that common carp in natural water may are influenced by the uneven distribution of food, seasonal changes, or environmental shifts, which impact their lipid metabolism, these findings warrant further research.

### 3.3. Cluster Analysis of Differential Metabolites

**Figure 5:**
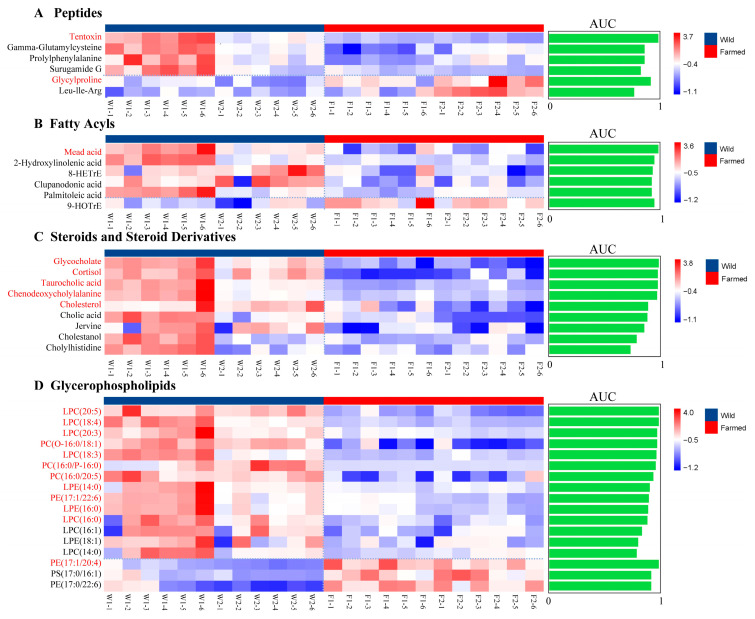
The heatmap categorizes metabolites into peptides (**A**), fatty acyls (**B**), steroids and steroid derivatives (**C**) and glycerophospholipids (**D**), with AUC values on the right. The left-side metabolites labeled in red (AUC > 0.9) indicate high significance, whereas the right green area represents AUC values. The red color of small lattice indicates high content and a blue color indicates low content.

Figure 5 shows the differences in metabolite abundance between wild and farmed common carp samples, which are divided into four categories: peptides (Figure 5A), fatty acyls (Figure 5B), steroids and steroid derivatives (Figure 5C), and glycerophospholipids (Figure 5D).

As shown in Figure 5A, the content of tentoxin and gamma-Glutamylcysteine in wild individuals is significantly higher, while the cultured population shows an increase in glycylproline concentration. At present, peptides metabolites have not been widely studied as biomarkers in agriculture. However, they have shown significant promise in medical applications. In medicine, numerous highly accurate peptides markers have been discovered. For example, natriuretic peptides can be used to predict future heart disease and mortality risk in healthy people [59]. Further research utilizing tandem mass tag (TMT) labeling combined with LC-MS/MS analyzed differential peptides between primary colorectal cancer specimens and matched adjacent epithelial tissues, establishing a new biomarker approach for Colorectal cancer (CRC) detection and treatment [60]. Peptides substances have shown accuracy in some medical identifications, demonstrating their potential as identification agents. Although their current application in agriculture is limited, we can attempt to apply them to the identification of wild-farmed common carp, but experimental verification is still needed.

The content of fatty acyls are presented in Figure 5B. The wild group exhibited predominance of metabolites such as Mead acid, clupanodonic acid and 2-hydroxylinolenic acid. The metabolites in the fatty acyls are widely thought to function as biomarkers in the identification of agricultural products and medicinal materials [61,62]. For example, C18:1 n-6c and C18:2 n-6c represent specific biomarkers for distinguishing organic farm and retail milk [63]. Similarly, 12-methyltridecanoic acid can be used as a biomarker for identifying different production areas of *Cordyceps sinensis* [64]. Although research on the use of fatty acyls to differentiate wild and farmed common carp is limited, several highly distinguishable candidate substances can be selected for further study. In the present study, Mead acid showed significant differences between the wild and farmed group (AUC > 0.9). Mead acid is synthesized from oleic acid under essential fatty acid deficiency (EFAD) conditions [65]. This suggests that wild common carp populations may face less consistent food availability, resulting in a deficiency of essential fatty acids and the accumulation of Mead acid. Further research could attempt to use Mead acid as a biomarker.

In this study, several steroid and steroid derivative metabolites with high discriminatory power were identified, including cortisol, taurocholic acid, and cholesterol, as well as other metabolites (AUC > 0.9) (Figure 5C). Cortisol is a well-known stress hormone that participates in various homeostatic maintenance actions, such as blood pressure, immune system, anti-inflammatory actions, and protein metabolism [66]. Taurocholic acid, as a natural bioactive substance found in animal bile, is used to treat a wide range of inflammatory diseases [67]. Cholesterol plays an essential role in cell membrane synthesis and in cell growth and differentiation [68]. It is speculated that the environmental instability of the wild habitat may subject wild common carp to increased stress, which, coupled with skin damage and infection, can lead to inflammation, resulting in the accumulation of these substances. In conclusion, future research should validate these candidate markers and develop rapid and reliable detection methods to identify wild and farmed common carp, thereby combating illegal fishing and assisting in the implementation of the Yangtze River fishing ban.

Figure 5D presents the analysis results of glycerophospholipids. Glycerophospholipids metabolites have the potential to serve as identification biomarkers. In a study, their analysis revealed significant lipid alterations: sphingomyelins and glycerophosphoserines (PSs) had decreased concentrations, while glycerophosphoinositols (PIs), glycerophosphoethanolamines (PEs), and glycerophosphocholines (PCs) showed elevated levels, suggesting potential biomarker development [69]. Previous studies have shown that there are metabolic differences between wild and farmed fish, which may be caused by differences in water quality, diet quality, and physical activity. These studies suggest that these classes of metabolites are anticipated to contribute significantly to the field of identification. Moreover, the differential metabolites identified in this study, such as prolylphenylalanine, Surugamide G, LPC(20:5), LPC(18:4), show significant differences between wild and farmed populations. Therefore, the different contents of these substances in wild and farmed common carp are likely to originate from their different environmental conditions.

### 3.4. Characteristic Metabolites of Different Regions

**Figure 6:**
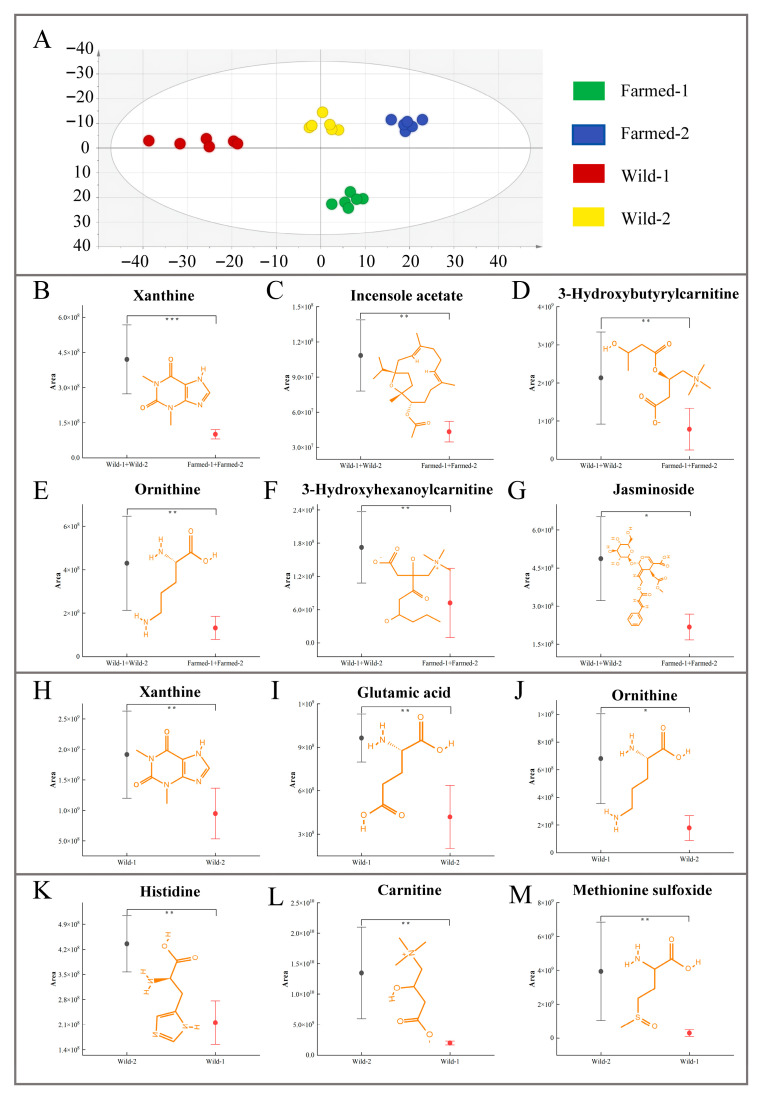
An OPLS-DA model to distinguish between wild and farmed common carps from four different regions (**A**). Comparative analysis between wild populations (wild-1 and wild-2) and farmed populations (farmed-1 and farmed-2) identified six significant differential metabolites (**B**–**G**), with wild-1 (**H**–**J**) and wild-2 (**K**–**M**) each showing three distinct metabolites. The asterisk denotes a statistically significant difference, with significance levels indicated as follows: * *p* < 0.05, ** *p* < 0.01, and *** *p* < 0.001.

To conduct an initial investigation into the potential differences in metabolite composition across various sections of the Yangtze River and to identify prospective metabolic biomarkers for source tracing, OPLS-DA analysis was applied to sample groups from each sampling site. Figure 6A presents the OPLS-DA analysis results of metabolites from the wild and farmed groups (wild-1, wild-2, farmed-1, and farmed-2). The analysis showed a distinct distribution pattern between wild and farmed samples, indicating significant regional differences in common carp metabolism. This provides the theoretical basis for subsequent screening of the dominant metabolites in distinct regions.

To ensure the accuracy of the identified differential metabolites, a two-step validation process was implemented. First, a comprehensive and comparative analysis between wild (wild-1 and wild-2) and farmed samples (farmed-1 and farmed-2), yielding six significant differential metabolites based on the criteria of VIP > 1, FC ≥ 2 and *p* < 0.05 (Figure 6B–G). Additionally, further comparisons were conducted between the wild groups (wild-1 versus wild-2) to identify potential markers for traceability. in the present study the content of 3 metabolites in the wild-1 samples was significantly higher than that in the wild-2 samples (Figure 6H–J). Moreover, compared with the wild-1 samples, 3 metabolites with significantly increased content were detected in the wild-2 samples (Figure 6K–M).

As shown in Figure 6B,H, there was a significant difference in xanthine content between the wild samples (wild-1 and wild-2) and the farmed samples (farmed-1 and farmed-2) (*p* < 0.001). Moreover, within the wild population, xanthine level in wild-1 was significantly higher than in wild-2 (*p* < 0.001), indicating that xanthine can not only serve as a potential marker for distinguishing wild and farmed groups, but may also be a characteristic metabolite of the wild-1 samples. Functionally, xanthine and its derivatives are key intermediates in the production of GMP, GDP, and GTP in cells that depend on a salvage pathway that recycles intermediates of degradation back into GTP and nucleic acids [70]. Its high level may reflect the more active nucleotide turnover and higher energy metabolism requirements of the wild carp (especially wild-1), which might be an adaptive response to the fluctuations in the wild environment. Notably, ornithine exhibited a similar trend (Figure 6E,J), with significant differences observed between the wild and farmed populations (*p* < 0.01) as well as within the wild population (*p* < 0.05). Ornithine plays a crucial role in the ornithine-urea cycle (OUC) [71]. Previous studies have shown that this cycle is a metabolic pathway through which certain fish maintain low ammonia levels in their bodies and prevent ammonia poisoning, such as *Protopterus aethiopicus* and *Protopterus annectens* [72]. In aquaculture, feed and additives are the main sources of ammonia, leading to excessive ammonia in aquaculture water bodies, which has become a common environmental problem [73]. The change in ornithine is likely due to elevated ammonia levels in the aquaculture water, which can affect OUC and potentially lead to ammonia poisoning. This finding is consistent with previous research results: ammonia is easily reached toxic levels in aquaculture environments, excessive exposure to ammonia leads to ammonia excretion disorders, and even causes death of aquatic animals [74].

The distribution of 3-hydroxybutyrylcarnitine and 3-hydroxyhexanoylcarnitine content exhibits significant differences between wild and farmed carp (*p* < 0.01; refer to Figure 6D,F). 3-hydroxybutyrylcarnitine, a product of pyruvate and carnitine, is an acylcarnitine and serves as a marker of mitochondrial function, particularly for fatty acid β-oxidation. It primarily facilitates fatty acid β-oxidation and their transport into mitochondria [75], while decrease 3-hydroxyhexanoylcarnitine levels indicate impaired fatty acid oxidation capacity [76]. The differences of 3-hydroxybutyrylcarnitine between the wild and farmed groups may be caused by the environment, the fatty acid β-oxidation in the wild group is stronger than that in the farmed group. Currently, in aquaculture, high-fat feed is often used as a substitute for high-quality protein to reduce costs, which leads to lipid metabolic problems in farmed fish generally, and their fatty acid β-oxidation capacity needs to be improved [77]. This is consistent with our speculation. Based on these findings, 3-hydroxybutyrylcarnitine and 3-hydroxyhexanoylcarnitine can be regarded as potential biomarkers for differentiating between wild and farmed common carp.

Statistically significant differences (*p* < 0.01) were observed in incensole acetate (IA) and other compounds when comparing wild and farmed common carp (Figure 6C). As shown in Figure 6G, the expression level of jasminoside exhibited significant differences (*p* < 0.05). Both of these substances are natural plant components: IA is a component of frankincense tree resin [78], while jasminoside is found in jasmine flowers [79]. The factors driving the accumulation of these substances in the wild fish remain unclear. The vegetation near the natural water environment is richer than that in the aquaculture environment [80]. It can be imagined that they are then synthesized by natural plants and may enter the water environment through processes such as erosion by rainwater, eventually accumulating in fish bodies through the food chain of wild fish.

The observed metabolic disparities between wild and farmed common carp are likely attributable to fundamental differences in their environmental conditions and nutritional sources. The farmed environment is characterized by controlled settings and standardized, high-fat commercial feeds, which can lead to elevated ammonia levels in water and altered lipid metabolism in fish [81,82]. Conversely, wild common carp reside in intricate and dynamic natural ecosystems characterized by abundant and diverse food sources. Research indicates that environmental factors substantially influence the composition of fish microbiota, especially in natural habitats where the gut microbiota of fish demonstrate greater diversity and complexity [83]. This diversity in diet and the need to constantly adapt to environmental variables (e.g., temperature fluctuations, predation pressure) likely drive the more active energy metabolism, stronger ammonia detoxification, and enhanced fatty acid β-oxidation observed in wild individuals, as reflected by metabolites like xanthine, ornithine, and acylcarnitines.

To assess the robustness of the six selected characteristic metabolites for regional discrimination, hierarchical clustering analysis was conducted on metabolites that showed significant differences between the wild-1 and wild-2 groups. The analysis revealed high intra-group consistency, with samples within each group clustering closely together, whereas samples from different groups were distinctly separated in the dendrogram (Figure S3). These findings indicated that a comprehensive analysis of the metabolic profiles of these six metabolites could effectively differentiate between the two regions at a preliminary level.

As shown in Figure 6I, there was a significant difference (*p* < 0.001) in the content of glutamic acid (Glu) between wild-1 and wild-2. Glu is a non-essential metabolic versatile amino acid and plays an essential role in the development of the central nervous system [84]. Studies have shown that for Atlantic salmon (*Salmo salar*), glutamate is an important substrate for the ammonia detoxification mechanism in the nervous system [85]. The potential conservation of this metabolic function in common carp, however, is presently unknown and warrants subsequent investigation. This may be related to the presence of a large number of submerged aquatic plants in the wild-2. Submerged aquatic plants absorb free ammonia (NH_4_^+^) in the water body and convert it into organic nitrogen (such as amino acids, proteins), thereby reducing the toxicity of ammonia to aquatic organisms [86]. In contrast, the ecological environment of the wild-1 may contain more free ammonia (NH_4_^+^), and the ammonia detoxification mechanism in the body of common carp is stronger, so it exhibits significantly different Glu levels from wild-2. In the present study, histidine, carnitine, and methionine sulfoxide exhibited significantly higher abundance in wild-2 (Figure 6K–M). Among them, histidine is an essential amino acid for fish requiring dietary intake [87]. Carnitine is a low-molecular-weight compound, mainly synthesized from essential amino acids methionine and lysine in the diet [88], while methioninesulfoxide is the main oxidation product of methionine [89]. This may be because the wild-2 area has a rich variety of aquatic plants and a complex zooplankton community structure, providing diverse food choices for grass carp. It may also have a unique nutritional structure that can provide a richer source of histidine and methionine. The identification of these region-specific metabolites not only provides a reliable basis for fish tracing the origin but also reveals the impact of environmental differences in the Yangtze River Basin, such as variations in nutritional structure and phytoplankton.

Previous research has established that the metabolite composition of fish is modulated by a multitude of factors, including environmental conditions, food resources, and physiological status, in both aquaculture environments and natural aquatic ecosystems [90,91,92,93]. In the current study, notable metabolic disparities were observed between two wild fish populations, despite their distribution in geographically adjacent natural waters, indicating that local micro-environmental variations may exert a significant influence. For instance, wild-2 area, with its abundant submerged plants, likely possesses a superior capacity for natural water purification, absorbing excess nitrogen and maintaining lower ammonia levels [94]. This could explain the lower demand for an internal ammonia detoxification mechanism (such as Glu) in wild-2 fish [95]. Conversely, the wild-1 area might be subject to greater environmental stress, potentially from anthropogenic sources such as agricultural runoff or domestic sewage, introducing higher levels of ammonia nitrogen [96]. This would necessitate a stronger physiological response for ammonia detoxification in wild-1 common carp. These environmental divergences may be associated with climatic conditions, precipitation patterns, and seasonal fluctuations in water quality. This suggests that the physicochemical properties of natural water bodies exert a direct influence on the metabolic profiles of wild populations [97,98,99,100].

### 3.5. Comprehensive Analysis of Water Factors, Dietary Pattern and Metabolomics

We conducted an analysis of water quality across various regions to examine the factors contributing to metabolic differences in fish populations. This study seeks to elucidate the impact of environmental factors on the metabolism of common carp. The findings are anticipated to offer a robust foundation for promoting the sustainability of fishery resources and the conservation of water resources within the Yangtze River Basin.

Table S1 presents our measurements of Ca, Mg, Cu, NH_4_-N, and TP in the water environment. The analysis revealed significant differences in Ca, Mg, NH_4_-N, and TP levels between wild and farmed groups (*p* < 0.001). This discovery is consistent with broader research that highlights the distinct environmental impacts with aquaculture practices. This study found that fish farming areas exhibited higher concentrations of nutrients like total phosphorus [101]. Similarly, the study highlights the complex interactions between aquaculture effluents and nutrient dynamics, particularly the role of ammonium and nitrate in influencing environmental outcomes [102]. In aquaculture, the administration of feed and pharmaceuticals can substantially influence the concentrations of minerals, including Ca and Mg, within the aquatic environment [103].

**Figure 7:**
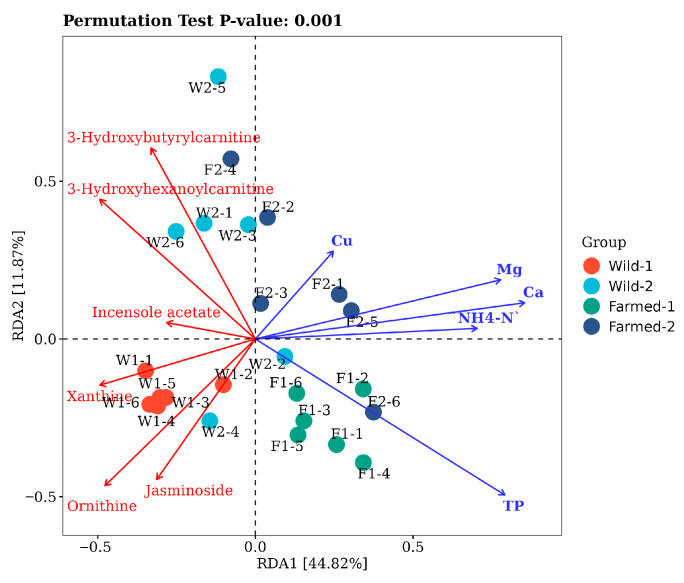
RDA of specific metabolites (red line) in the Yangtze River basin and farmed areas and water environmental factors (blue line).

Figure 7 presents the RDA successfully passed the permutation test, yielding a *p*-value of 0.001, which signifies a statistically significant relationship between environmental variables and sample distribution. The interpretation of correlation within RDA plot is contingent upon the angular relationship between the arrows: a positive correlation is indicated by an acute angle (less than 90°), while a negative correlation is represented by an obtuse angle (greater than 90°) [104].

Figure 7 shows the red line for 3-hydroxybutyrylcarnitine and the blue line for Cu converging at an acute angle, indicating a positive correlation. This relationship in common carp warrants further study for validation. Cu, a common water pollutant, may induce oxidative stress and alter liver transcriptomes in common carp, affecting lipid metabolism [105]. This stress may relate to 3-hydroxybutyryl carnitine, crucial for fatty acid oxidation. While copper is essential for the growth and immune function of common carp, dietary copper nanoparticles (Cu-NPs) enhance growth and immune responses, leading to increased copper accumulation [106], possibly linked to the metabolic role of 3-hydroxybutyryl lipid [76]. In addition, the relationship between ornithine and NH_4_-N is characterized by an obtuse angle, suggesting a negative correlation between the two variables. The metabolic pathways of ammonia nitrogen (NH_4_-N) in plants have elucidated its intricate association with ornithine. Research indicates that in plants utilizing NH_4_^+^ as the principal nitrogen source, the reduction of the diazotization isotope ^15^N is correlated with NH_4_^+^/NH_3_ toxicity. This isotope fractionation phenomenon implies that NH_4_^+^ uptake and metabolism may impact the nitrogen equilibrium within plants, thereby indirectly affecting ornithine metabolic pathways [107]. In conclusion, these alterations in nitrogen isotopes may be linked to the processes of ornithine synthesis and degradation. Integrating these research findings facilitates a more nuanced understanding of the intricate relationship among carp cultivated in diverse regions, environmental factors, and metabolic regulation.

Furthermore, research indicates substantial differences in dietary between wild and farmed fish, which in turn influence their physiology and lead to distinct metabolic characteristics [108]. A study conducted in aquaculture ponds revealed that farm-raised common carp preferentially consume diets of higher biochemical quality [109], in contrast to the natural food sources available to their wild counterparts [110]. In summary, dietary differences in aquaculture environments could affect the metabolite profiles of fish.

## 4. Conclusions

Using UHPLC-MS/MS-based metabolomics, this study identified significant metabolic differences between wild and farmed common carp. PCA and OPLS-DA analyses confirmed a clear separation between groups (R^2^Y = 0.947, Q^2^ = 0.874). We identified 16 potential biomarkers (AUC > 0.9), including phytosphingosine and succinic acid in farmed fish as well as cytidine, uridine, and mead acid in wild fish which may be linked to high-density farming, environmental instability and dietary variability. Through geographical tracing analysis, our study identified biomarkers in different sampling areas, such as Glu and Carnitine, which may be associated with environmental differences in different sections of the Yangtze River. RDA suggests that Cu concentration in water may impact 3-hydroxybutyryl enrichment, while NH_4_-N concentration may affect ornithine enrichment. These findings offer traceability biomarkers that effectively differentiate between wild and farmed carp, thereby contributing to efforts aimed at combating illegal fishing. Future research will aim to precisely identify and functionally validate the targeted substances, while also systematically collecting and analyzing samples of both wild and farmed carp, to further enhance the translational and applied value of the work.

## Data Availability

The data not included in the manuscript are available upon reasonable request to the corresponding authors.

## Supplementary Materials

The following supporting information can be downloaded at: https://www.mdpi.com/article/10.3390/biology14121695/s1, Figure S1. Correlation plot of merged positive and negative ion mode QC samples; Figure S2. Hierarchical clustering analyse comparing farmed and wild groups. The farmed group is designated as F1 and F2, whereas the wild group is classified as W1 and W2; Figure S3. Hierarchical clustering analyse comparing wild-1 and wild-2 groups; Table S1. Analysis of differences between wild and farmed aquatic environmental factors.
