# Supplementary material for: Nutritional Quality Fingerprinting of Wild and Farmed Cyprinus carpio: A UHPLC-MS/MS-Based Traceability Strategy
Source: Biology (Basel). 2025 Nov 28;14(12):1695. doi: 10.3390/biology14121695 (PMC12730570; doi:10.3390/biology14121695)
Supplement: Supplementary file 1 [file biology-14-01695-s001.zip › biology-3934890-supplementary.pdf]

Supplementary Materials

Supporting files:

Table S1

Figure S1-S3

Table S1. Analysis of differences between wild and farmed aquatic environmental factors.

Factor	Wild		Farmed		Wild	Farmed
	Wild-1	Wild-2	Farmed-1	Farmed-2		
Cu /ug/L	0.26±0.01^{c}	0.25±0.01^{c}	0.81±0.04^{b}	1.12±0.14^{a}	0.82± 0.10	0.97 ± 0.19
Ca /ug/L	1.56±0.01^{d}	2.12±0.04^{c}	5.75±0.10^{b}	8.47±0.27^{a}	1.84 ± 0.31	7.11 ± 1.50***
Mg /ug/L	1.39±0.02^{c}	1.85±0.03^{c}	5.95±0.08^{b}	11.24±0.25^{a}	1.62 ± 0.25	8.60 ± 2.91***
NH_4-N /mg/L	0.16±0.02^{c}	0.12±0.01^{d}	0.28±0.03^{b}	0.43±0.02^{a}	0.14 ± 0.03	0.36 ± 0.09***
TP /mg/L	0.15±0.03^{c}	0.10±0.01^{c}	0.97±0.06^{a}	0.86±0.55^{b}	0.17 ± 0.03	0.91 ± 0.08***

Different letters indicate significant regional differences ($P < 0.05$).

*** indicates that there is a difference between the two regions ($P < 0.001$).

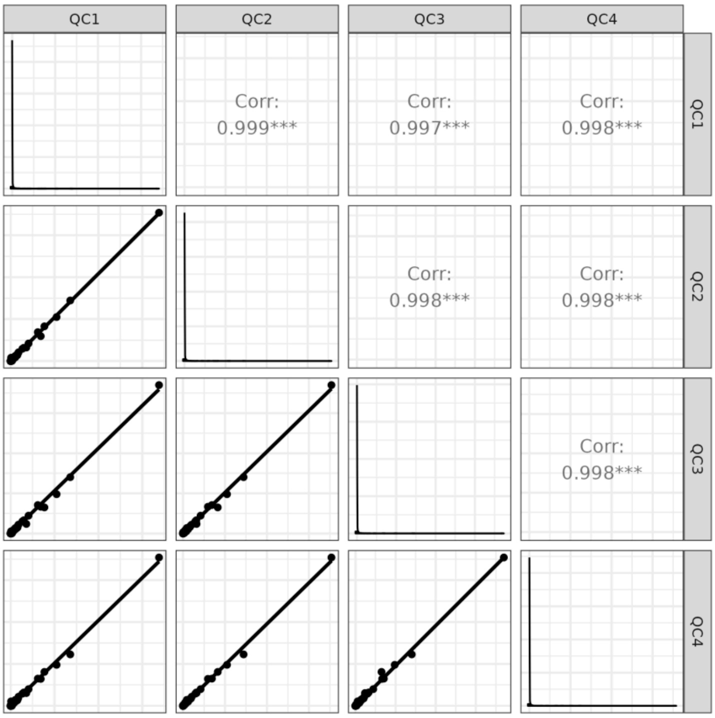

Figure S1. Correlation plot of merged positive and negative ion mode QC samples.

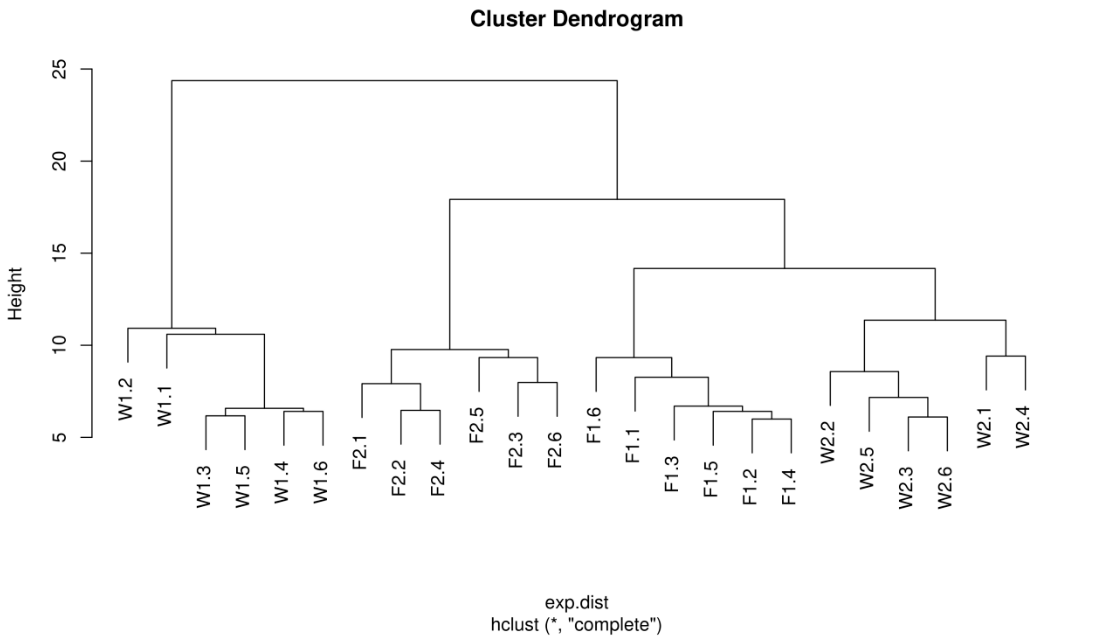

Figure S2. Hierarchical clustering analyse comparing farmed and wild groups. The farmed group is designated as F1 and F2, whereas the wild group is classified as W1 and W2.

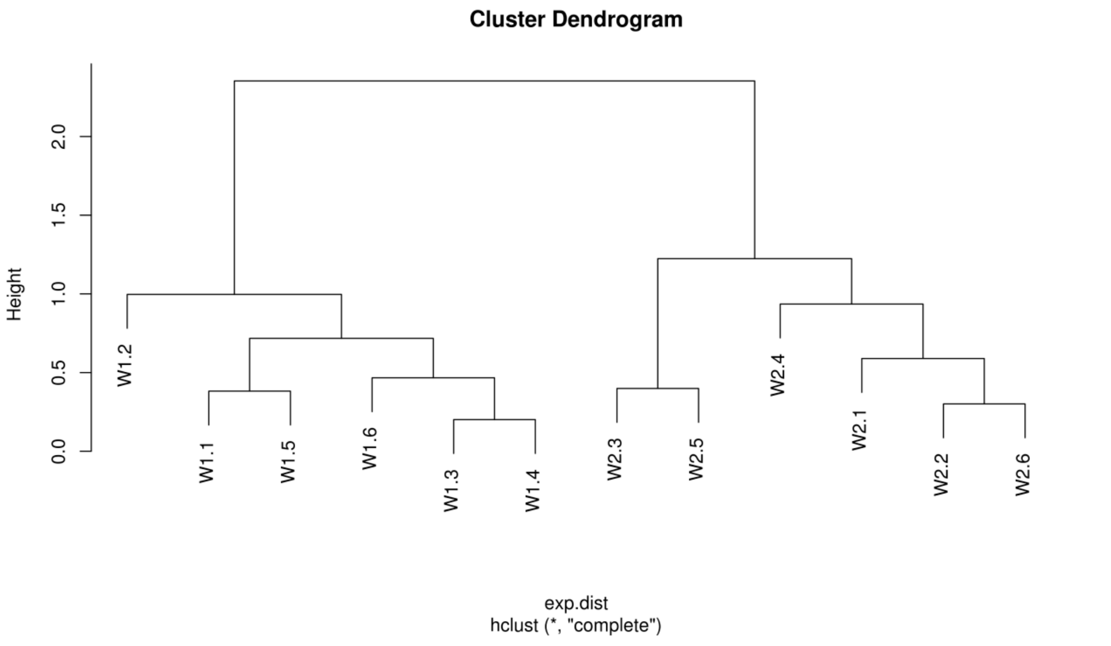

Figure S3. Hierarchical clustering analyse comparing wild-1 and wild-2 groups.
